# Anti-aging effect of extracellular vesicles from mesenchymal stromal cells on senescence-induced chondrocytes in osteoarthritis

**DOI:** 10.18632/aging.206158

**Published:** 2024-11-22

**Authors:** Jérémy Boulestreau, Marie Maumus, Giuliana Bertolino Minani, Christian Jorgensen, Danièle Noël

**Affiliations:** 1 IRMB, University of Montpellier, INSERM, Montpellier, France; 2 Department of Rheumatology, Clinical Immunology and Osteoarticular Disease Therapeutic Unit, CHU de Montpellier, Montpellier, France

**Keywords:** mesenchymal stromal cell, extracellular vesicle, senescence, aging, osteoarthritis, regenerative medicine

## Abstract

Age is the most important risk factor for degenerative diseases such as osteoarthritis (OA). It is associated with the accumulation of senescent cells in joint tissues that contribute to the pathogenesis of OA, in particular through the release of senescence-associated secretory phenotype (SASP) factors. Mesenchymal stromal cells (MSCs) and their derived extracellular vesicles (EVs) are promising treatments for OA. However, the senoprotective effects of MSC-derived EVs in OA have been poorly investigated. Here, we used EVs from human adipose tissue-derived MSCs (ASC-EVs) in two models of inflammaging (IL1β)- and DNA damage (etoposide)-induced senescence in OA chondrocytes. We showed that the addition of ASC-EVs was effective in reducing senescence parameters, including the number of SA-β-Gal-positive cells, the accumulation of γH2AX foci in nuclei and the secretion of SASP factors. In addition, ASC-EVs demonstrated therapeutic efficacy when injected into a murine model of OA. Several markers of senescence, inflammation and oxidative stress were decreased shortly after injection likely explaining the therapeutic efficacy. In conclusion, ASC-EVs exert a senoprotective function both *in vitro*, in two models of induced senescence in OA chondrocytes and, *in vivo*, in the murine model of collagenase-induced OA.

## INTRODUCTION

Osteoarthritis (OA) is the most common joint disorder characterized by cartilage degradation, bone changes including osteophytes and subchondral sclerosis and, low-grade synovitis. Several risk factors have been identified namely obesity, repetitive traumas, metabolic disorders and genetics but the most prevalent one is age. Aging is a complex process resulting from the accumulation of unpredictable molecular and cellular changes and nine hallmarks: genomic instability, telomere attrition, epigenetic alteration, loss of proteostasis, metabolic dysfunction, mitochondrial dysfunction, stem cell exhaustion, cellular senescence and altered intercellular communication [1, 2]. Cellular senescence has been associated with OA and is described by permanent cell cycle arrest with resistance to cell death by necrosis, apoptosis or autophagy [3]. Senescent cells release a secretome containing various bioactive molecules, the so-called senescence-associated secretory phenotype (SASP). These molecules can be released in extracellular vesicles (EVs), which participate in cell-to-cell communication, and are involved in disease propagation [4]. In late OA, failure of repair responses due to senescence would lead to progressive degeneration of cartilage [5]. However, the exact mechanism linking senescence and OA pathology remains unclear.

Among the therapeutic strategies being investigated for the treatment of OA is the use of mesenchymal stromal cells (MSCs). They have demonstrated therapeutic efficacy in preclinical models of OA by attenuating inflammation and reducing cartilage and bone lesions, as well as improving pain and functional parameters in the clinic [6, 7]. More recently, EVs released by MSCs have been reported to be the main mediators of the beneficial effects of the parental cells. They exert a protective effect on OA chondrocytes *in vitro* and in mouse models of OA, and promote osteochondral regeneration [8–10]. The beneficial effect of MSC-EVs was associated with a lower rate of apoptosis and a regulation of genes involved in cartilage homeostasis [8, 11–13]. However, little is known about the role of EVs in the senescence of chondrocytes or other cells of the joint compartment involved in the initiation and progression of OA.

The first demonstration that adipose-derived mesenchymal stromal cell EVs (ASC-EVs) can down-regulate senescence features in OA was made on IL1β-treated human osteoblasts isolated from patients with advanced OA [14]. The authors reported decreased SA-β-Gal activity, accumulation of γH2AX foci and production of inflammatory mediators in osteoblasts. In another study, the conditioned medium from ASCs was able to downregulate senescence markers in primary OA chondrocytes subjected to inflammatory stress [15]. Very recently, antler ASC-EVs were shown to reduce the accumulation of senescent cells in the cartilage of mice induced to develop OA [16]. In this model of anterior cruciate ligament transection, the number of p16-positive cells was lower in the cartilage of EV-treated mice and was correlated with the improvement in OA score and inversely correlated with the number of proliferating cells. In addition to these studies, it has been reported that umbilical cord- or embryonic stem cell-derived EVs and ASC-EVs can alleviate cellular aging in bone marrow-derived MSCs [17–19]. To our knowledge, only one recent study has reported the anti-aging effect of umbilical cord MSC-EVs engineered to express an affinity peptide on OA chondrocytes [20].

It is widely accepted that aging and inflammation, or inflammaging, are important contributors to the development of OA [21]. There are complex links between inflammation, senescence, DNA damage and the impact on many diseases [22]. However, the processes that trigger senescence are different. Persistent inflammation, even of low grade, could be either a cause or a consequence of the senescence that is observed in OA. To date, models of senescence have not adequately addressed the respective roles of inflammation or age-related accumulation of DNA damage in chondrocytes. Furthermore, the effect of native MSC-EVs on chondrocyte senescence stimulated by different pro-aging environments has not been investigated. In this context, the IL1β-induced chondrocyte model is a well-described model of inflammation-induced senescence mediated by the SASP whereas DNA damage-induced senescence is poorly described [23].

Our main objective is to evaluate the senomorphic properties of ASC-EVs. We performed *in vitro* studies on primary OA chondrocytes using the IL1β-induced senescence model of inflammation and developed an etoposide-induced DNA damage model of senescence. We then evaluated the ASC-EVs in the mouse model of collagenase-induced OA.

## RESULTS

### Characterization of etoposide-induced senescent chondrocytes

**Figure 1:**
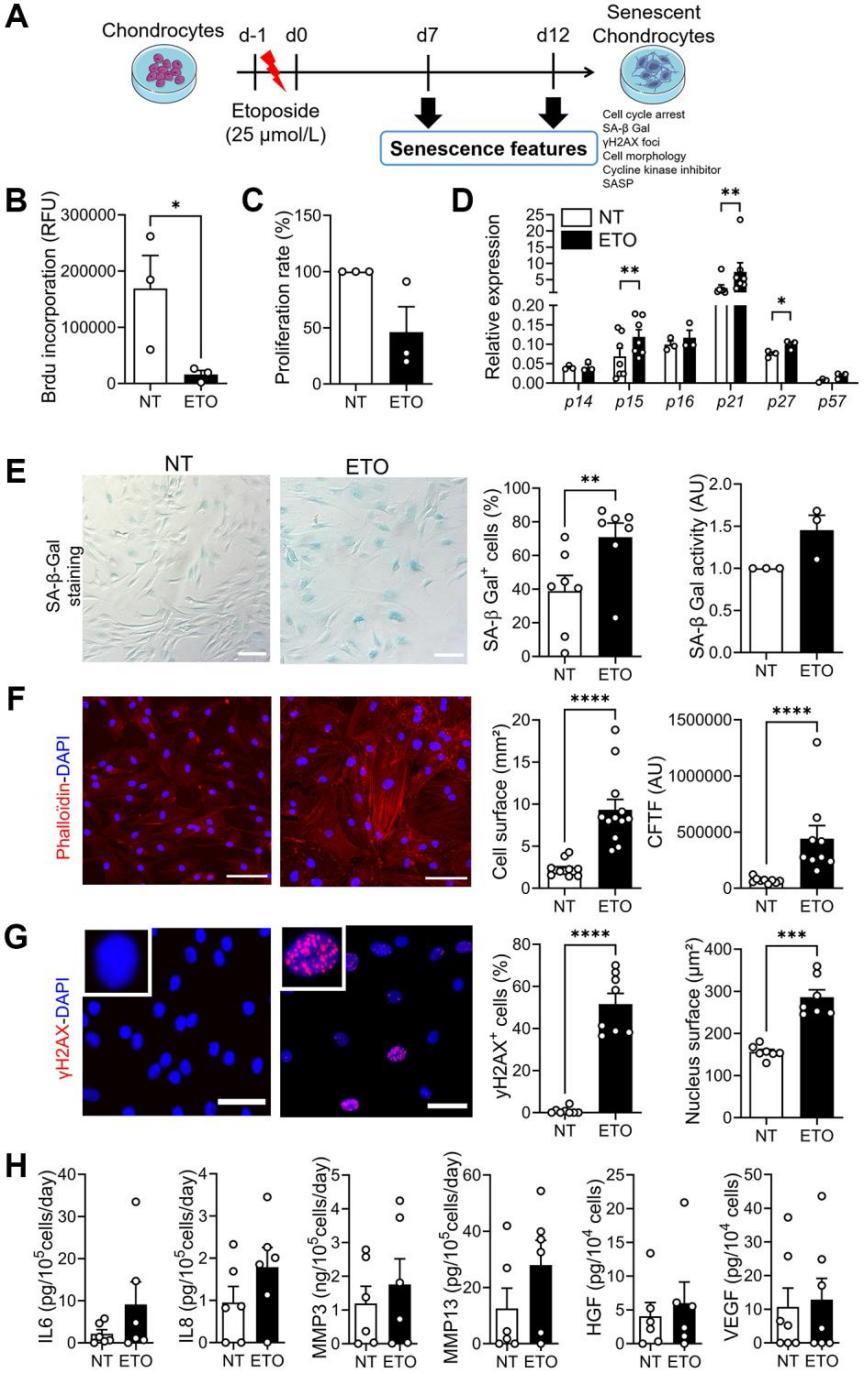
**Characterization of etoposide-induced senescence in human chondrocytes at day 7.** (**A**) Schematic workflow of etoposide (ETO)-induced senescence in osteoarthritic chondrocytes. (**B**) Level of BrdU incorporation in non-treated (NT) and ETO-treated chondrocytes (*n* = 3). (**C**) Percentage of proliferation in NT and ETO chondrocytes (*n* = 3). (**D**) Relative expression of Cyclin-Dependent Kinase Inhibitors in NT and ETO chondrocytes by RT-qPCR (*n* = 3–7). (**E**) Representative pictures of SA-β-Galactosidase (Gal) staining in NT and ETO chondrocytes (left panel; bars: 100 µm). Percentage of SA-β-Gal-positive cells (*n* = 7) and SA-β-Gal activity quantified by fluorometry (*n* = 3) (right panel). (**F**) Representative staining of actin stress fibers (in red) and nuclei (in blue) with Phalloidin and DAPI, respectively (left panel; bars: 100 µm). Quantification of the cell surface (*n* = 12) and corrected total cell fluorescence (CTCF) (*n* = 9) (right panels). (**G**) Representative pictures of γH2AX-positive foci (red spot) in DAPI stained nuclei (blue) (left panel; bars: 50 µm). Percentage of cells with γH2AX-positive nuclei (*n* = 8) and quantification of nucleus surface (*n* = 7) (right panel). (**H**) Protein secretion in supernatants of NT and ETO chondrocytes quantified by ELISA (*n* = 6). Data are shown as mean ± SEM. Statistical analysis used the Mann-Whitney test (**B**, **D**: pairwise comparisons, **E**: left panel, **F**, **G**, **H**) or Wilcoxon signed rank test (**C**, **E**: right panel). ^*^p < 0.05, ^**^p < 0.01, ^***^p < 0.001, ^****^p < 0.0001.

We generated a model of DNA damage-induced senescence model by incubating human chondrocytes isolated from OA donors with ETO for 24 hours and cultured them for 7 or 12 days (Figure 1A). As early as day 7, ETO-treated chondrocytes stopped proliferating, as indicated by low levels of BrdU incorporation and low proliferation rate compared with non-treated (NT) chondrocytes (Figure 1B, 1C). Simultaneously, the expression levels of three major cyclin-dependent kinase inhibitors (CDKI), p15INK4b (p15), p21cdkn1a (p21) and p27cdkn1b (p27) were increased (Figure 1D). SA-β-Gal staining revealed an increase in the number of SA-β-Gal-positive cells and SA-β-Gal activity in ETO-treated chondrocytes (Figure 1E). The presence of stress fibers was observed by Phalloidin staining, which also revealed the increased cell surface (Figure 1F). The ETO treatment induced DNA damage foci as indicated by the appearance of γH2AX positive nuclei in chondrocytes and the increase in nuclear surface area (Figure 1G). Finally, we evaluated the secretory profile of ETO-treated chondrocytes and observed a tendency to increased SASP (Figure 1H). Similar senescence features were also observed at day 12 confirming that we have generated a stable model of DNA damage-induced senescence in human OA chondrocytes (Supplementary Figure 1).

### Senoprotective effect of ASC-EVs on etoposide-induced senescent chondrocytes

**Figure 2:**
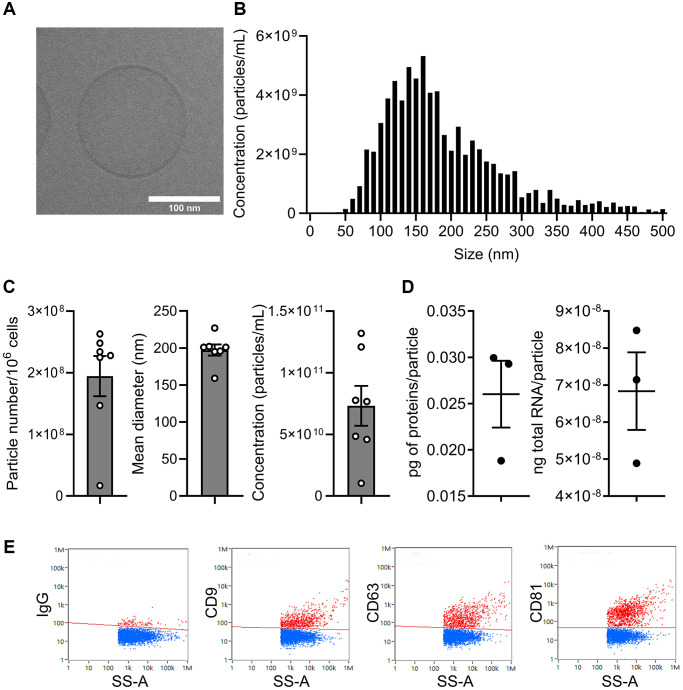
**Characterization of extracellular vesicles isolated from ASCs.** (**A**) Representative picture of one single EV by cryo-transmission electron microscopy (scale bar: 100 nm). (**B**) Size distribution of EVs (one representative sample). (**C**) Number of particles produced by 10^6^ ASCs per day, mean size and particle concentration after isolation (*n* = 7). (**D**) Quantity of total proteins (left panel) and total RNA (right panel) contained per particle (*n* = 3). (**E**) Expression profile of tetraspanin markers on the surface of EVs by nanofcm analysis.

We then evaluated the effect of ASC-EVs on the prevention of senescence in OA chondrocytes. EVs were prepared from ASCs and characterized according to the recommendations of the International Society on Extracellular Vesicles [24]. The lipid bilayer structure of EVs was confirmed by cryo-TEM analysis (Figure 2A) and the size distribution of EVs is shown in Figure 2B. The number of EVs produced by 10^6^ ASCs was 1.93 ± 0.3 × 10^8^ EVs per day, with a mean size of 197 ± 6.8 nm, resulting in a final concentration of 7.3 ± 1.6 × 10^10^ EVs/mL after isolation (Figure 2C). The total protein and RNA content was approximately 0.026 ± 0.006 pg/particle and 6.32 ± 1.38 × 10^−8^ ng/particle, respectively (Figure 2D). The presence of markers characteristic for EVs (CD9, CD63, CD81) was evaluated by nanoflow cytometry (Figure 2E).

**Figure 3:**
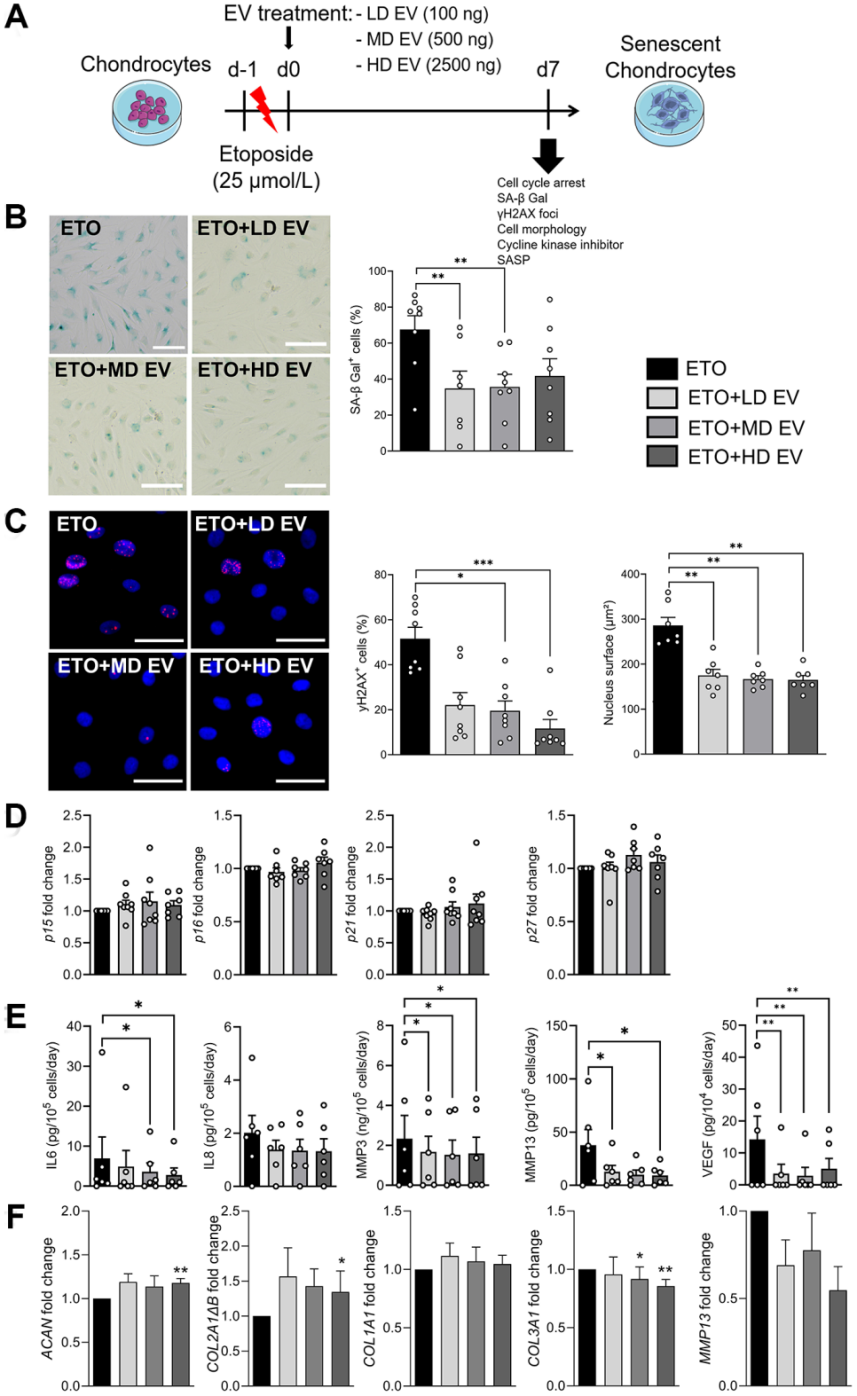
**Senoprotective effect of extracellular vesicles from ASCs on etoposide-induced senescent chondrocytes.** (**A**) Schematic workflow of etoposide (ETO)-treated human chondrocytes cultured with different doses of EVs isolated from ASCs: Low dose (LD), medium dose (MD) and high dose (HD). (**B**) Representative pictures of SA-β-Gal staining in human chondrocytes (left panel; bars: 50 µm) and percentage of SA-β-Gal positive cells (right panel) (*n* = 7, 8). (**C**) Representative pictures of γH2AX foci (red) in the nuclei (blue) of chondrocytes (left panel; bars: 50 µm). Percentage of cells with γH2AX foci in nuclei and quantification of nucleus surface (*n* = 7, 8) (right panels). (**D**) Relative expression of Cyclin-Dependent Kinase Inhibitors in chondrocytes (*n* = 6, 7). (**E**) Protein secretion in supernatants of ETO-treated chondrocytes quantified by ELISA (*n* = 6, 7). (**F**) Relative expression of markers in ETO-treated chondrocytes (*n* = 6). Data are shown as mean ± SEM. Statistical analysis used the Mann-Whitney test (**B**, **C**) or the Wilcoxon signed rank test (**D**, **F**) or the Wilcoxon matched pair signed rank test (**E**) for pair-wised comparisons versus the ETO group. ^*^p < 0.05, ^**^p < 0.01, ^***^p < 0.001.

Different doses of ASC-EVs were added to ETO-treated chondrocytes on day 0 as shown (Figure 3A). The effect of each dose of EVs was compared with the ETO control. At day 7, the number of SA-β-Gal-positive chondrocytes was lower in the presence of EVs, regardless of the dose (Figure 3B). In addition, the number of γH2AX-positive nuclei of ETO-treated chondrocytes was lower when incubated with EVs (Figure 3C). We also observed enlarged nuclei in ETO-treated chondrocytes, which were smaller when EVs were added. The addition of EVs did not affect the expression of CDKIs (Figure 3D). We also found that EVs decreased the secretion of IL6, MMP3, MMP13 and VEGF (Figure 3E). Finally, EVs upregulated the anabolic markers of chondrocytes *ACAN* and *COL2A1ΔB* and downregulated *MMP13* and *COL3A1*, the catabolic and fibrotic markers of OA chondrocytes, respectively (Figure 3F). Thus, ASC-EVs can prevent the senescence of OA chondrocytes exposed to DNA damage by regulating the balance between their anabolic and catabolic activities.

### IL1β-induced senescence in OA chondrocytes

**Figure 4:**
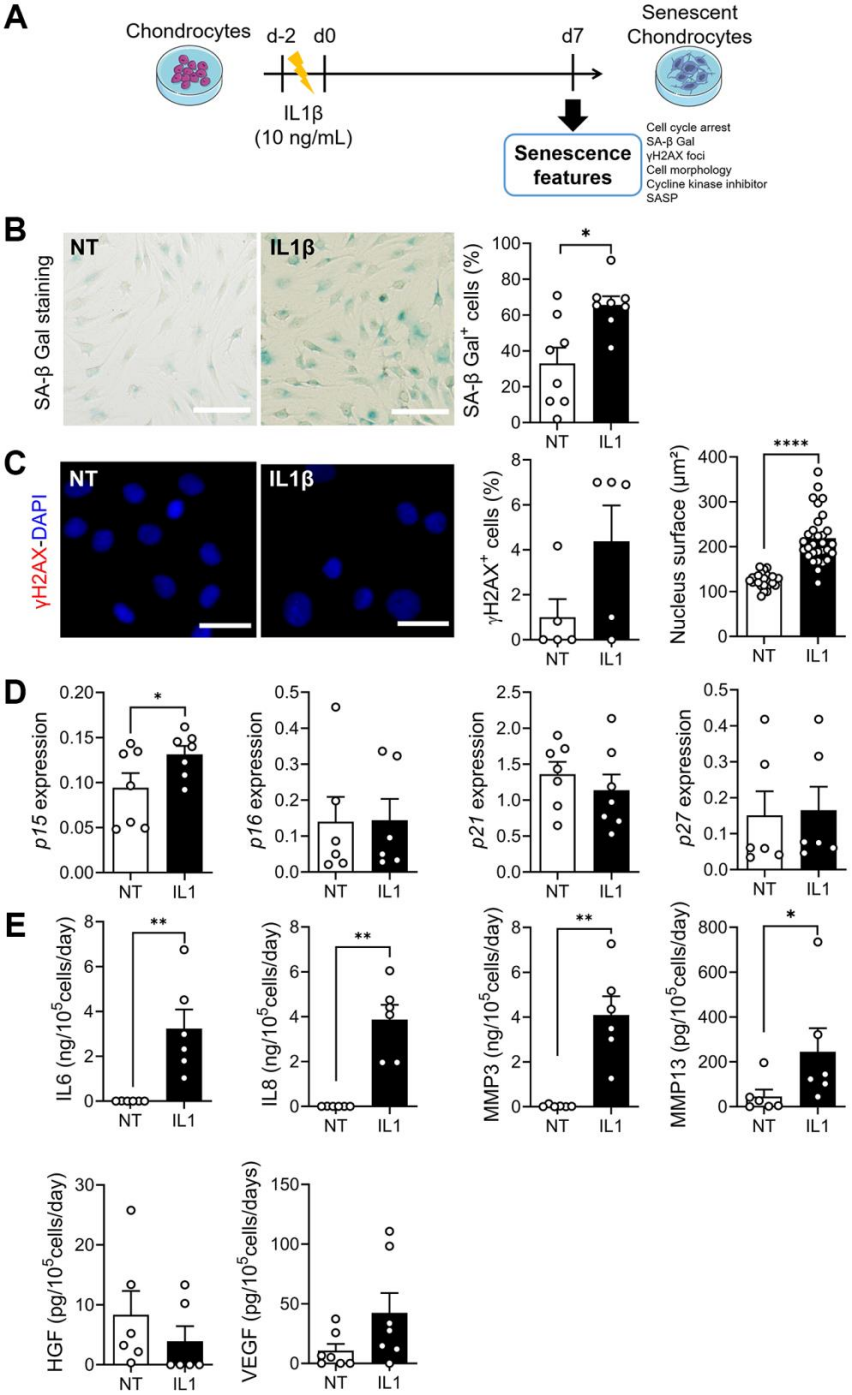
**Model of IL1β-induced senescence in chondrocytes.** (**A**) Schematic workflow of IL1β-induced senescence in chondrocytes. (**B**) Representative pictures of SA-β-Gal staining in non-treated (NT) and IL1β-treated human chondrocytes (left panel; bars: 50 µm) and percentage of SA-β-Gal positive cells (right panel) (*n* = 7, 8). (**C**) Representative pictures of chondrocytes with γH2AX foci (red) in nuclei (blue). (Left panel; bars: 100 µm). Percentage of chondrocytes with γH2AX foci (*n* = 5) and quantification of nucleus surface (*n* = 30) (right panels). (**D**) Relative expression of Cyclin-Dependent Kinase Inhibitors in chondrocytes (*n* = 6, 7). (**E**) Protein secretion in supernatants of IL1β-treated chondrocytes quantified by ELISA (*n* = 6). Statistical analysis used the Mann-Whitney test for all panels except for the nucleus surface graph where an unpaired *t*-test was used. ^*^p < 0.05, ^**^p < 0.01, ^****^p < 0.0001.

To determine whether the beneficial effect of ASC-EVs was specific for DNA damage-induced senescence, we used a model of inflammation-induced senescence using IL1β, as previously described [25] (Figure 4A). Although a number of OA chondrocytes already stained positive for SA-β-Gal under control conditions, the addition of IL1β increased the number of SA-β-Gal-positive chondrocytes (Figure 4B). IL1β treatment did not induce DNA damage as evidenced by the low number of nuclei with γH2AX-positive foci (Figure 4C). However, the nuclear surface area, which is indicative of morphological changes associated with senescence, was increased. The expression levels of most CDKIs were unchanged in IL1β-treated chondrocytes, except for *p15INK4b*, which was upregulated (Figure 4D). Importantly, we found a huge increase of SASP factors, namely inflammatory (IL6, IL8) and catabolic (MMP3, MMP13) factors and the growth factor VEGF (Figure 4E). Therefore, compared to ETO-induced senescence, IL1β-induced senescent chondrocytes are characterized by a lack of DNA damage and an inflammatory SASP.

### Senoprotective effect of ASC-EVs on IL1β-induced chondrocytes

**Figure 5:**
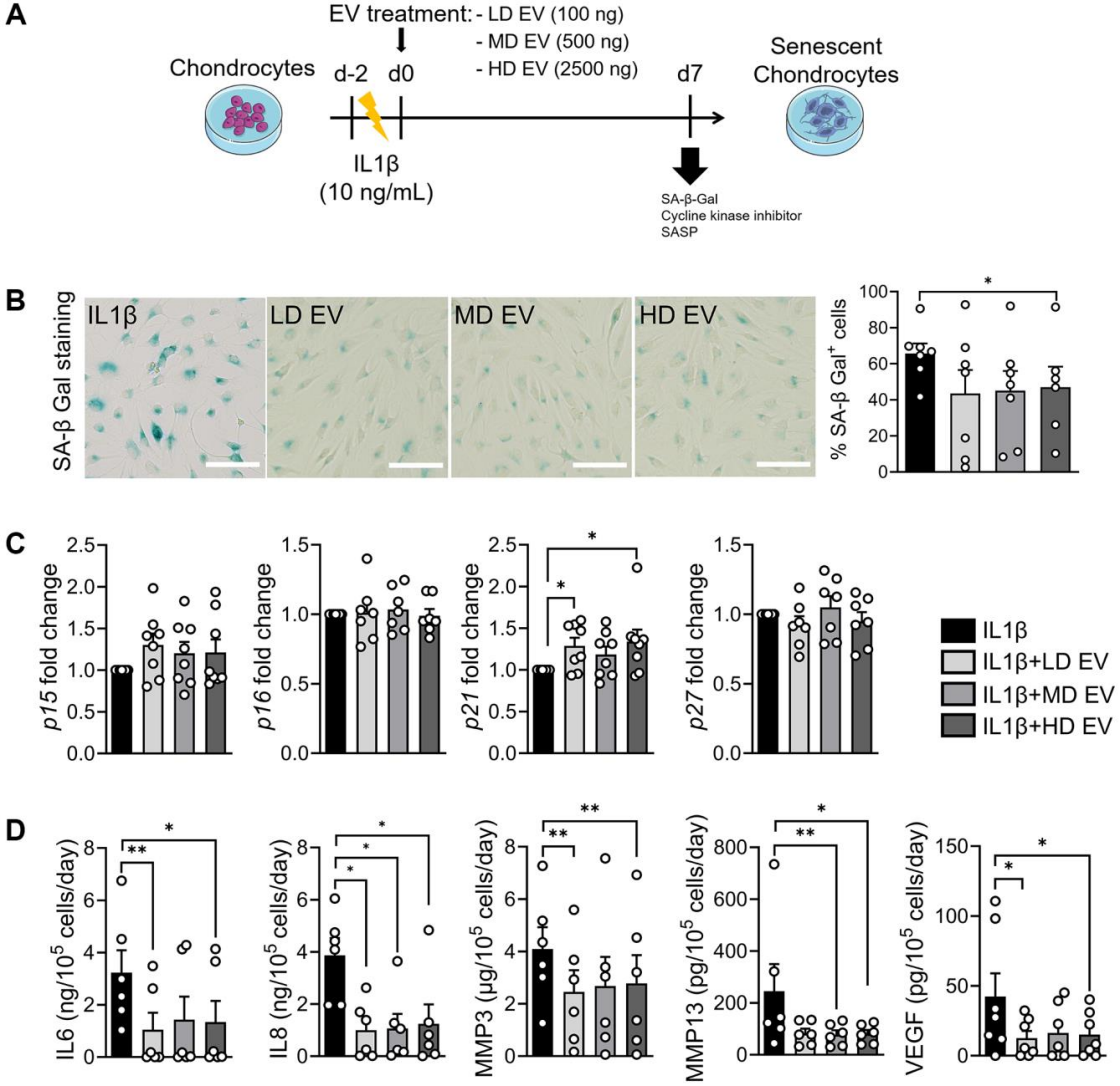
**ASCs-derived extracellular vesicles exert a senoprotective effect on IL1β-induced senescence in chondrocytes.** (**A**) Workflow of IL1β-induced senescence in primary human chondrocytes and treatment with different doses of ASCs-derived EVs: Low dose (LD), medium dose (MD) and high dose (HD). (**B**) Representative pictures of SA-β-Gal staining in chondrocytes (left panel; bars: 100 µm) and percentage of SA-β-Gal positive cells (*n* = 6, 7) (right panel). (**C**) Relative expression of Cyclin-Dependent Kinase Inhibitors in IL1β-treated chondrocytes (*n* = 6, 7). (**D**) Protein secretion in supernatants of IL1β-treated chondrocytes quantified by ELISA (*n* = 6). Data are shown as mean ± SEM. Statistical analysis used the Mann-Whitney test (**B**, **C**) or the Wilcoxon signed rank test (**C**) or the Wilcoxon matched pair signed rank test (**D**) for pair-wised comparisons versus the IL1β group. ^*^p < 0.05, ^**^p < 0.01.

IL1β-induced senescent chondrocytes were incubated with different doses of EVs as described (Figure 5A). We compared the effect of each dose of EVs with the IL1β control group. At day 7, we did not examine γH2AX staining because DNA damage is not induced in this model but the percentage of SA-β-Gal-positive chondrocytes was lower when EVs were added (Figure 5B). The expression of CDKIs was not altered by the addition of EVs, except for *p21* which was increased (Figure 5C). The secretion of IL6, IL8, MMP3, MMP13 and VEGF was lower when EVs were added (Figure 5D). Although *FOXO1* was not regulated in IL1β-treated chondrocytes upon addition of EVs, the expression of *FOXO3* was higher suggesting a regulatory effect on autophagy-related genes (data not shown). Thus, the senoprotective effect of ASC-EVs was demonstrated in this model of inflammation-induced chondrocyte senescence.

### Early changes in gene expression after administration of ASC-EVs protect joints from OA development

**Figure 6:**
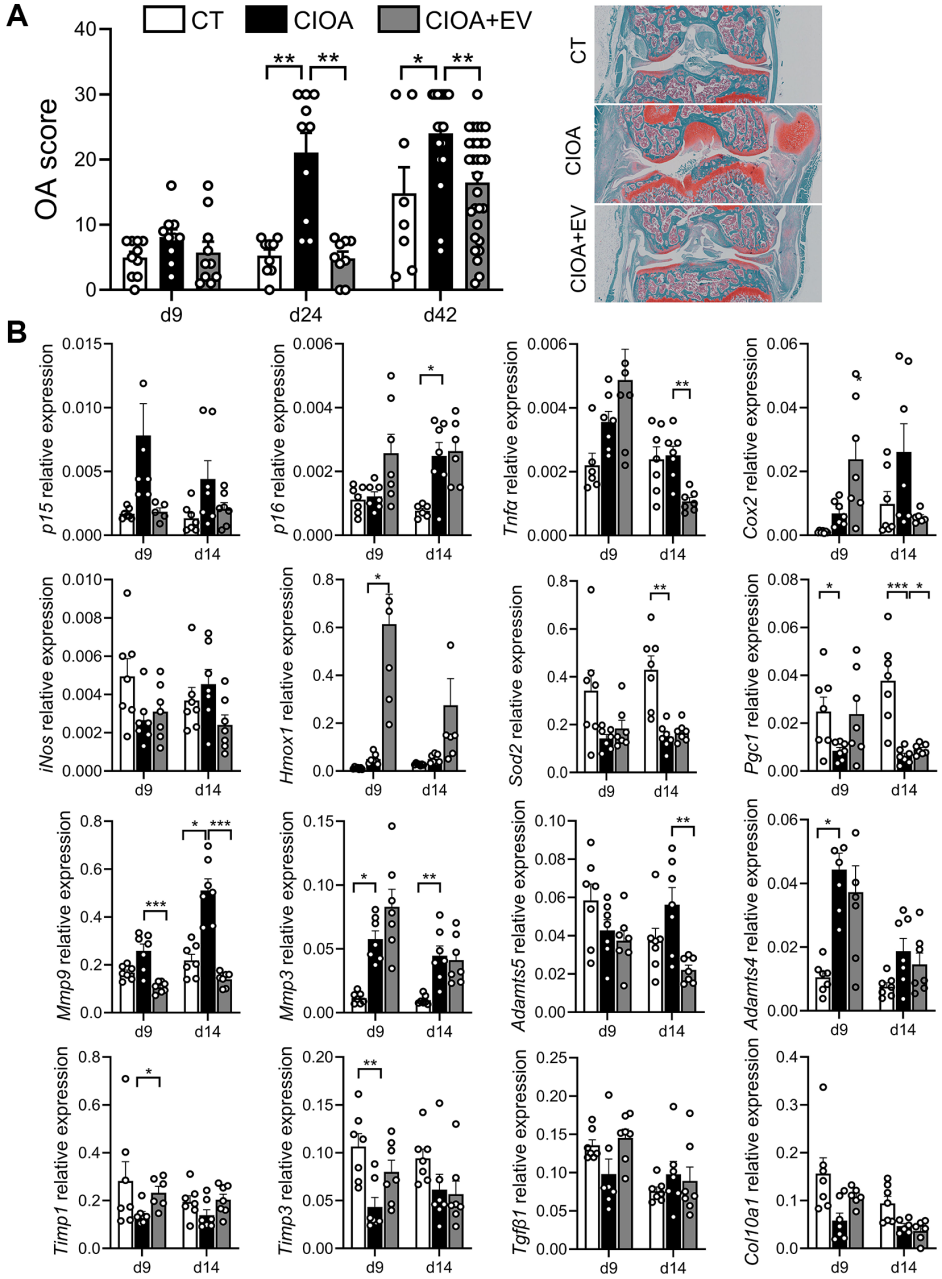
**ASCs-derived extracellular vesicles exert a therapeutic effect in OA mice.** (**A**) Histological OA score determined at different time points in collagenase-induced osteoarthritis (CIOA) mice (left panel). Representative pictures of histological sections from each group of mice at day 42 (*n* = 5/group). (**B**) Relative expression of genes representative of senescence, inflammation, oxidative stress and metabolism in the joints of CIOA mice (*n* = 6, 7/group). Data are shown as mean ± SEM. Statistical analysis used Kruskall-Wallis test with a Dunn’s post-test. ^*^p < 0.05, ^**^p < 0.01, ^***^p < 0.001.

We have previously shown that murine BM-MSC-EVs can protect mice from OA and reduce cartilage and bone changes in the CIOA model [8]. In the same model, we also showed that the peak of *p16* expression and several SASP factors (*Il1β, Il6, Mmp13*) is observed at day 14, while the p16 activity is the highest at day 24 after CIOA induction [26]. Therefore, the effect of administration of ASC-EVs (approximately 3 × 10^8^ particles in 5 µL) has been tested in mice with CIOA at early time points: at day 9 and 14 for gene expression and, at day 9, 24 and 42 for histological analysis, as the peak of protein expression is delayed compared to the peak of RNA expression. Histologic analysis revealed that injection of EVs reduced the OA score at day 24 and day 42 (Figure 6A). EVs also tended to downregulate the expression of *p15* at day 9 and day 14 while *p16* expression was not regulated (Figure 6B). The expression of the inflammatory mediators *Tnfα, Cox2, iNos* tended to be higher in EV-treated OA joints at day 9 and to strongly decrease at day 14 while the anti-inflammatory factors *Hmox-1, Sod2* and *Pgc1* tended to be increased both at both days 9 and 14. In addition, the expression of catabolic markers *Mmp9* and* Adamts5* was downregulated by ASC-EVs at day 9 and day 14, whereas the expression of *Mmp* inhibitors *Timp1, Timp3* and anabolic markers *Tgfβ1* and *Col10A1* tended to be upregulated at day 9. Collectively, the results indicate that ASC-EVs profoundly influence several factors that are early dysregulated in OA joints, reducing senescence markers and balancing the inflammatory/anti-inflammatory and anabolic/catabolic activities to restore homeostasis.

## DISCUSSION

We demonstrate that ASC-EVs exert senoprotective effects in the two models of inflammation- and DNA damage-induced senescent chondrocytes. Furthermore, a single injection of ASC-EVs can regulate the early expression of several senescence- and OA-associated factors in the *in vivo* OA model thereby restoring joint homeostasis and preventing OA-associated changes.

Cellular senescence increases with age and is a hallmark of OA, where the accumulation of senescent chondrocytes correlates with disease severity [25]. Oxidative stress, overloading, inflammation, and DNA damage can induce chondrocyte senescence. In OA, levels of DNA damage have been reported to be higher than in age-matched “healthy” donors [27]. Persistent DNA damage can be induced by various stressors, particularly exposure to genotoxic agents, which activate the DNA damage repair (DDR) signaling and consequently, upregulate the p53/p21 pathway. DNA damage-induced chondrocyte senescence can be induced by several factors, including irradiation and chemotherapeutic drugs such as cisplatin or doxorubicin [28–31]. In the present study, we used etoposide to induce the senescence of OA chondrocytes. Two recent studies have reported similar results on some of the features of senescence (proliferation arrest, SA-β-Gal-positive cell number and IL6 or p53 increase) using chondrocyte cell lines [32, 33]. In contrast to these studies, we used primary human OA chondrocytes, which are likely primed *in vivo* by the inflammatory and senescent environment of the injured joint but are still responsive to a genotoxic agent, and are more relevant for evaluating a treatment response. The most common model of chondrocyte senescence is induced by inflammation, specifically exposure to IL1β [34]. Comparing the two models of etoposide- and IL1β-induced senescence in our study, we observed several similar features but also some differences were noticed, notably low levels of γH2AX positive nuclear foci, the induction of a single CDKI p15 and upregulation of SASP factors in IL1β-treated chondrocytes. Nevertheless, ASC-EVs displayed senoprotective functions in both models suggesting that they can regulate multiple stress responses that are induced by different stimuli.

Several studies have reported the interest of stem cell-derived EVs in exerting anti-aging effects (for review, see [35]). Indeed, MSC-EVs have been shown to inhibit the production of reactive oxygen species (ROS), protect fibroblasts from cell cycle arrest, decrease cell senescence, and increase the expression of antioxidant enzymes in models of UVB-induced dermal fibroblast photoaging [36, 37]. In inflammatory diseases, the inhibition of inflammation is a critical mechanism of the anti-aging effect of MSC-EVs. Inhibition of NF-κB/TNFα signaling by the lncRNA MALAT1 present in MSC-EVs was found to prevent cardiomyocyte aging [38]. Induction of macrophage polarization toward the M2 phenotype was also shown to prevent cardiac damage via S1P/SK1 signaling [39]. Another important parameter to prevent cell senescence is the donor age of the MSCs used for the production of EVs. Several studies have reported that MSC-EVs from young donors can rejuvenate aging cells [19, 40, 41]. However, few studies have described a direct senoprotective effect of MSC-EVs in OA (for review, see [4]). MSC-EVs have been shown to prevent the induction of senescence in OA osteoblasts but a single recent report has provided evidence that umbilical cord-derived MSC-EVs can rejuvenate OA chondrocytes [20]. To our knowledge, our study is the first to demonstrate that EVs isolated from ASCs can exert a senoprotective function in two models of senescence-induced OA chondrocytes by ameliorating several senescence-associated characteristics. The most important finding of our study was to establish that one intra-articular injection of ASC-EVs can profoundly affect the joint environment soon after administration. This early regulation of senescence- and OA-related markers was sufficient to maintain joint homeostasis and prevent cartilage degradation in the long term, at least for the five weeks documented.

Chondrocytes have been shown to effectively take up MSC-EVs, in both 2D and 3D microenvironments, including in cartilage explants, suggesting that *in vivo* uptake of MSC-EVs is likely to occur [42–44]. In addition to their senoprotective effects, MSC-EVs exert other beneficial effects on OA chondrocytes, particularly on the induction of proliferation and migration and, the reduction of apoptosis and inflammation, as described in several studies [8, 45, 46]. MSC-EVs can also stimulate the production of extracellular matrix components in chondrocytes when they are preconditioned or when they overexpress chondrogenic factors, including miR-140-5p, miR-210 and miR-92a [8, 45–48]. However, similar to our data, native MSC-EVs exert a significant but weak anabolic effect on chondrocyte markers (ACAN, COL2A1, COL1A1, SOX9), while the anti-catabolic and anti-inflammatory effects are more potent [42, 46]. Therefore, ASC-EVs represent an interesting therapeutic option to modulate the biological processes that are deregulated in OA. Several mediators, in particular miRNAs, have been proposed to play a key role in the therapeutic efficacy of ASC-EVs by acting on different processes in the different joint compartments in OA (for review, see [49]). Overexpression of these mediators in EVs through parental cell preconditioning or engineering could further enhance their functional effects. Finally, we demonstrated that a single injection of EVs has rapid and long-lasting effects *in vivo* but it may be interesting to evaluate multiple injections at several weeks intervals to prolong their efficacy as it will likely be required for OA patients.

A possible limitation of the present study is that we used a population of EVs that were isolated by differential ultracentrifugation and we cannot exclude that part of the therapeutic activity is related to the non-EV fraction. However, we have previously reported that the immunosuppressive function of total EVs is mediated by the fraction of EVs and not the soluble fraction [50]. Although we have not investigated the mechanism of action of EVs, we have recently identified proteins that are differentially loaded in healthy and senescent ASC-derived EVs [51]. A number of these proteins are related to the regulation of inflammation and senescence. Unraveling the mechanism of action of these proteins in the context of OA may help to develop engineered EVs with more potent functions. In conclusion, we have provided evidence that ASC-EVs exert a senoprotective effect on OA chondrocytes using two models of induced senescence, which likely contribute to the therapeutic effect observed *in vivo* in OA mice. In addition to their anti-inflammatory and regenerative properties, our study confirms that ASC-EVs may be a relevant option for future clinical applications in degenerative diseases, such as OA, which are increasing with the population aging.

## MATERIALS AND METHODS

### Cells and culture media

ASCs were obtained from 7 healthy donors (aged 48.14 ± 5 years) from surgical residues obtained after aesthetic liposuction. Written informed consent was obtained from all subjects and approval was obtained from the French Ministry of Higher Education and Research (approval DC-2009-1052). The isolation and characterization of ASCs have been reported previously [52]. ASCs were expanded in α-MEM medium containing 10% fetal calf serum (FCS), 100 µg/mL penicillin/streptomycin (PS), 2 mM glutamine (Glu), and 1 ng/mL basic fibroblast growth factor (CellGenix, Freiburg, Germany). ASCs were used between passages 1 and 3.

Chondrocytes were isolated from OA patients undergoing total knee arthroplasty. Articular cartilage was harvested from the femoral condyles of patients (1 female and 6 males; mean age: 73.7 ± 2.1). Patient informed consent was obtained from the French Ministry of Research and Innovation (approval DC-2010-1185). For chondrocyte isolation, knee cartilage slices were incubated in 2.5 mg/mL pronase (Sigma-Aldrich, Saint-Quentin-Fallavier, France) for 1 h at 37°C followed by 2 mg/mL type II collagenase (Sigma) overnight at 37°C. The digested pieces were filtered through a 70 µm cell strainer and the cell suspension was cultured in DMEM with PS/Glu/Fungizone/10% FCS (proliferation medium) at the density of 25,000 cells/cm² until the end of passage 1.

### *In vitro* models of senescence-induced chondrocytes

For the DNA damage-induced senescence model, chondrocytes were plated at 10,000 cells/cm² in proliferative medium for 24 h and then incubated in presence or absence of etoposide (ETO; Sigma; 25 µM) for 24 h. Cells were washed three times with phosphate-buffered saline (PBS) and maintained in proliferative medium for seven or twelve days in culture. In the inflammation-driven model of senescence, chondrocytes were plated at 10,000 cells/cm² in proliferative medium for 24 h and incubated in the presence or absence of IL1β (Bio-Techne, Noyal Châtillon sur Seiche; 10 ng/mL) for 48 h. Cells were washed three times with PBS and cultured in proliferation medium for seven days in culture. At the end of the culture, chondrocytes were either fixed or collected and stored at -80°C. Supernatants were stored at −20°C.

### Cell proliferation assay

Chondrocytes were plated at low density (1 × 10^4^ cells/cm²) in a 6-wells plate in proliferative medium. After seven or twelve days, cells were trypsinized and the number of living cells was determined by the trypan blue exclusion test. Additionally, cell proliferation was quantified using the Cell Proliferation Elisa BrdU assay as described by the manufacturer (Roche Life Science, L’Isle-d’Abeau Chesnes, France).

### Senescence-associated β-galactosidase assay

Chondrocytes were fixed with 2.5% glutaraldehyde in PBS for 5 min at room temperature. After three washes in PBS, cells were incubated with a solution of 5 mM Potassium ferrocyanide, 5 mM Potassium ferricyanide, 200 mM citric acid/sodium phosphate buffer pH 6, 150 mM sodium Chloride, 2 mM magnesium chloride, 1 mg/mL of X-gal (Promega, Charbonnières-les-Bains, France) at 37°C for 4 h. Cells were examined and photographed under a microscope (EVOS M5000, Invitrogen, Illkirch, France). SA-β-Gal-positive cells were quantified using the cell counter complement of the ImageJ Software.

SA-β-Gal activity was assessed using the 96-well Cellular Senescence Activity Assay (Cell Biolabs, Clinisciences, Nanterre, France) following manufacturer’s instructions. Fluorescence was measured using a Varioskan Flash microplate reader (Thermo Fisher Scientific, Illkirch, France).

### Immunofluorescence assays

For γH2AX staining, chondrocytes were fixed with 3.7% formaldehyde in PBS for 15 min at room temperature. Blocking solution containing 5% normal goat serum and 0.3% Triton X-100 in PBS was added for 1 h at room temperature. After three washes in PBS, cells were incubated with an anti-phospho-histone H2AX (Ser139) antibody overnight at 4°C (Cell Signaling Technology, Sigma Aldrich, L’Isle-d’Abeau Chesnes, France). Finally, cells were incubated with Alexa Fluor 594 goat anti-rabbit IgG (H+L) (Thermo Fisher Scientific) and DAPI. Cells were examined under a microscope (EVOS M5000, Invitrogen) and photographed. γH2AX foci and nucleus area were quantified using the cell counter complement of the ImageJ Software.

For F-actin staining, chondrocytes were fixed with 3.7% formaldehyde in PBS and permeabilized with 0.1% TRITON X-100 in PBS. Cells were stained with a solution of 50 µg/mL Phalloidin-Tetramethyl-rhodamine B isothiocyanate (Phalloidin-TRITC; Sigma) for 40 min at room temperature, according to the supplier’s recommendations. The cells were then stained with DAPI and washed three times with PBS. Cells were examined under a microscope (EVOS M5000, Invitrogen) and photographed. ImageJ Software was used to quantify cell surface and the corrected total cell fluorescence (CTCF = integrated density - (area of selected cell × mean fluorescence of background readings)).

### RNA extraction and RT-qPCR

The total RNA was extracted using 350 µL RLT buffer from the RNeasy Mini Kit according to the supplier’s recommendations (Qiagen, Les Ulis, France). The reverse transcription of 500 ng RNA was obtained by incubation with the M-MLV reverse transcriptase (Thermo Fisher Scientific). Primers were designed using the Primer 3 software (Table 1) and synthesized by Eurofins Genomics (Ebersberg, Germany). Real-time PCR was done on 10 ng cDNA using the SYBR Green I Master mix (Roche Diagnostics, Meylan, France) or using Taqman Gene Expression assays (Thermo Fisher Scientific; Table 2). Values were normalized to the Ribosomal Protein S9 (RPS9) housekeeping gene and expressed as a relative expression or fold change using the respective formulae 2^−ΔCT^ or 2^−ΔΔCT^.

**Table 1:** List of primers for RT-qPCR.

**Gene (human)**	**Sequence forward**	**Sequence reverse**
***ACAN***	TCGAGGACAGCGAGGCC	TCGAGGGTGTAGCGTGTAGAGA
***COL2A1ΔB***	CAGACGCTGGTGCTGCT	TCCTGGTTGCCGGACAT
***COL1A1***	CCTGGATGCCATCAAAGTCT	CGCCATACTCGAACTGGAAT
***COL3A1***	CGCCCTCCTAATGGTCAAGG	AGGGCCTGAAGGACCAGCTT
***MMP13***	GACTTCCCAGGAATTGGTGA	TACCCCAAATGCTCTTCAGG
***p14ARF***	CCCTCGTGCTGATGCTACTG	ACCTGGTCTTCTAGG AAGCGG
***p15INK4b***	GACCGGGAATAACCTTCCAT	CACCAGGTCCAGTCAAGGAT
***p16INK4a***	GAAGGTCCCTCAGACATCCCC	CCCTGTAGGACCTTCGGTGAC
***p21cdkn1a***	AGGTGGACCTGGAGACTCTCAG	TCCTCTTGGAGAAGATCAGCCG
***p27KIP1***	ATAAGGAAGCGACCTGCAACCG	TTCTTGGGCGTCTGCTCCACAG
***p57KIP2***	GCGGCGATCAAGAAGCTGT	GCTTGGCGAAGAAATCGGAGA
***RPS9***	GATTACATCCTGGGCCTGAA	ATGAAGGACGGGATGTTCAC
**Gene (mouse)**	**Sequence forward**	**Sequence reverse**
***p16INK4a***	CGCAGGTTCTTCGTCACTGT	TGTTCACGAAAGCCAGAGCG
***tnfa***	AGCCCACGTCGTAGCAAACCA	TGTCTTTGAGATCCATGCCGTTGGC
***cox2***	CCAGCACTTCACCCATCAGTT	ACCCAGGTCCTCGCTTATGA
***inos***	CCTTGTTCAGCTACGCCTTC	GCTTGTCACCACCAGCAGTA
***hmox1***	GCAGAGCCGTCTCGAGCATA	GCATTCTCGGCTTGGATGTG
***sod2***	TCA GGA CCC ATT GCA AGG AA	TGT GGC CGT GAG TGA CGT TT
***pgc1***	AAA CTT GCT AGC GGT CCT CA	TGT TGA CAA ATG CTC TTC GC
***mmp9***	TCCAGTTTGGTGTCGCGGAGCACG	CAGGGGGAAAGGCGTGTGCCAGA
***mmp3***	CGATGATGAACGATGGACAGAGG	CTTGGCTGAGTGGTAGAGTCCCAG
***adamts5***	CTGCCTTCAAGGCAAATGTGTGG	CAATGGCGGTAGGCAAACTGC
***adamts4***	GAACGGTGGCAAGTATTGTGAGG	TTCGGTGGTTGTAGGCAGCACA
***timp1***	CTCCGCCCTTCGCATGGACATT	GGGGGCCATCATGGTATCTGCTCT
***timp3***	AGGATGCCTTCTGCAACTCCGA	GTGTAGACCAGAGTGCCAAAGG
***tgfβ1***	TGCGCTTGCAGAGATTAAAA	CTGCCGTACAACTCCAGTGA
***col10a1***	TGCTGCCTCAAATACCCTTT	CAGGAATGCCTTGTTCTCCT
***rps9***	GCTGTTGACGCTAGACGAGA	ATCTTCAGGCCCAGGATGTA

**Table 2:** TaqMan^®^ gene expression assay ID.

**Gene**	**ID**
***Human FOXO1***	Hs00231106_m1
***Human FOXO3***	Hs00818121_m1
***Mouse p15INK4b***	Mm07295536_m1

### ELISA assays

HGF, VEGF, MMP-3, MMP13, IL-6 and IL8 were quantified in the supernatants from cultures by using the respective Enzyme-linked immunosorbent assays (Bio-Techne, RnD Systems, Rennes, France).

### Production and isolation of EVs

ASCs were seeded at 2.5 × 10^3^ cells/cm^2^ and cultured for 4 days. The EV-free medium was obtained by recovering the αMEM medium containing 20% FCS after overnight ultracentrifugation at 100,000 × g at 4°C. This medium was then diluted to get the production medium containing 3% EV-free FCS. After one wash with PBS, ASCs were further cultured in the production medium for 72 h. Cells were eliminated from the conditioned supernatant by centrifugation at 300 g, 4°C for 10 min, while debris and apoptotic bodies were discarded by centrifugation at 2500 g, 4°C for 25 min. Total EVs were then pelleted by two ultracentrifugation steps at 100,000g, 4°C for 2 hours. EVs were characterized by their size, concentration by interferometric light microscopy (Videodrop, Myriade, Paris, France) and their structure by cryo-TEM. The presence of tetraspanins was analyzed by Nanoparticle Flow Cytometry (Nanofcm, Nottingham, UK) using the CD9-APC (clone MEM-61, 1:25, Abcam, UK), CD63-APC (clone MEM-259, 1:50, Invitrogen), CD81-APC (clone MEM-38, 1:100, Abcam) and isotypic control IgG1-APC (1:25, BD Biosciences, USA). Protein and RNA levels were quantified using the Micro BCA Protein Assay Kit (Thermo Fisher Scientific) and the RNeasy Micro Kit (Qiagen, Les Ulis, France), respectively. Freshly prepared ASC-EVs were used.

### Co-culture experiments

Chondrocytes were seeded at high density (5 × 10^4^ cells/cm²) in proliferative medium for 24 h. Cells were treated without or with etoposide (25 µM) for 24 h or IL-1β (10 ng/mL) for 48 h, as described above. Chondrocytes were then washed three times with PBS and cultured in presence of different doses of EVs (LD: low dose at 100 ng equivalent total proteins; MD: medium dose at 500 ng and HD: high dose at 2500 ng) and maintained for seven days in 3 mL of minimal medium (DMEM supplemented with PS, proline (0.35 mmol/L), ascorbic acid (0.17 mmol/L) and sodium pyruvate (1 mmol/L)). Chondrocytes were collected or fixed and supernatants were stored at −20°C.

### Collagenase-induced osteoarthritis model

The collagenase-induced OA (CIOA) mouse model was performed in 10-week-old male C57BL/6 mice provided by the Janvier Labs (France) as previously described [53] and in accordance with the guidelines and regulations of the Ethical Committee for Animal Experimentation of the Languedoc-Roussillon (approval APAFIS#5349-2016050918198875). Briefly, 5 µL of 1U type VII collagenase (CIOA) or saline (CT) were injected intra-articularly (IA) into the right knee joint of each mouse on days 0 and 2. On day 7, mice were randomized into three groups that received IA injections of EVs (250 ng/5 µL; CIOA+EV group) or 5 µL saline (CT and CIOA groups). The experimental unit for the experiment is a single animal and no data were excluded at the end of the study. Mice were euthanized on day 9 and 14 for gene expression analysis (*n* = 6/group; 36 in total) and, on day 9, 24 and 42 for histological analysis (*n* = 5/group; *n* = 45 in total). For gene expression analysis, the knee joints were harvested, mechanically dissociated and stored in Trizol (Thermo Fisher Scientific) at −80°C. For histology, hind paws were fixed in 3.7% formaldehyde for Safranin O/fast green staining. Modified Pritzker OARSI scoring was performed by two independent investigators who were blinded to the treatment groups as described [54].

### Statistical analysis

Statistical analyses were performed using the GraphPad 9 Prism Software. Data distribution was assessed using the Shapiro–Wilk normality test. The statistical tests used either the Mann-Whitney test or the unpaired *t*-test for non-parametric or parametric data pair-wise comparisons, respectively. The Wilcoxon signed rang test was used for the comparison of non-parametric data to a normalized value or the Wilcoxon matched pair signed rang test for paired non-parametric data comparison. The tests used for each panel are indicated in the figure legend.

## Supplementary Materials

Supplementary Figure 1
