# Supplementary material for: Anti-aging effect of extracellular vesicles from mesenchymal stromal cells on senescence-induced chondrocytes in osteoarthritis
Source: Aging (Albany NY). 2024 Nov 22;16(21):13252–70. doi: 10.18632/aging.206158 (PMC11719114; doi:10.18632/aging.206158)
Supplement: Supplementary Figure 1 [file aging-16-206158-s001.pdf]

## SUPPLEMENTARY FIGURE

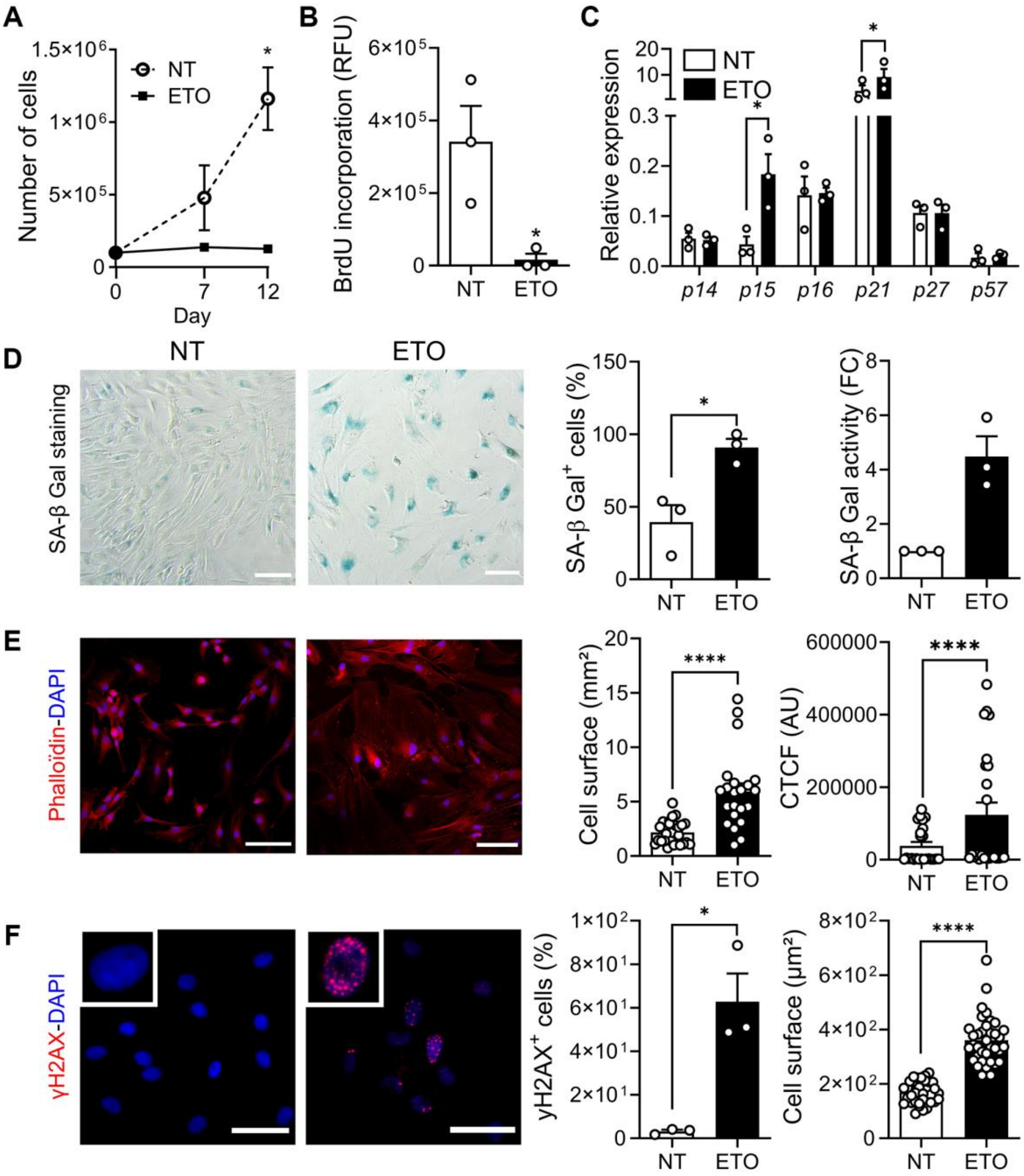


**Supplementary Figure 1. Characterization of etoposide-induced senescence in human chondrocytes at twelve days.** (**A**) Cumulative number of cells in non-treated (NT) and etoposide (ETO)-treated human OA chondrocytes ($n$ = 3, 4). (**B**) Level of BrdU incorporation in NT and ETO-treated chondrocytes at day 12 ($n$ = 3). (**C**) Relative expression of Cyclin-Dependent Kinase Inhibitors in chondrocytes ($n$ = 3). (**D**) Representative pictures of SA-β-Gal staining in NT and ETO chondrocytes (left panel). Percentage of SA-β-Gal positive cells ($n$ = 3) and SA β-Gal activity quantified by fluorometry ($n$ = 3) (right panels). (**E**) Staining of actin stress fibers with phalloidin (red) and nuclei with DAPI (blue). Bars: 100 µm (left panel). Quantification of cell surface ($n$ = 22–30) and corrected total cell fluorescence (CTCF) ($n$ = 24) (right panels). (**F**) Representative pictures of nuclei stained with DAPI (blue) and γH2AX-positive foci in chondrocytes (red). Bars: 50 µm (left panel). Percentage of chondrocytes with γH2AX foci in nuclei ($n$ = 3) and quantification of nucleus surface ($n$ = 36) (right panels). Data are shown as mean ± SEM. Statistical analysis used the Mann-Whitney test (**A**–**D** and **F**: left panel) or the Wilcoxon signed rank test (**D**: right panel) or the unpaired $t$-test (**E**, **F**: right panel), for pair-wised comparisons versus the NT group. $^{*}p < 0.05$, $^{****}p < 0.0001$.
